# Melatonin Alleviates Contrast-Induced Acute Kidney Injury by Activation of Sirt3

**DOI:** 10.1155/2021/6668887

**Published:** 2021-05-25

**Authors:** Chunmei Zhang, Mengying Suo, Lingxin Liu, Yan Qi, Chen Zhang, Lin Xie, Xuehui Zheng, Chang Ma, Jingyuan Li, Jianmin Yang, Peili Bu

**Affiliations:** The Key Laboratory of Cardiovascular Remodeling and Function Research, Chinese Ministry of Education, Chinese National Health Commission and Chinese Academy of Medical Sciences, The State and Shandong Province Joint Key Laboratory of Translational Cardiovascular Medicine, Department of Cardiology, Qilu Hospital, Cheeloo College of Medicine, Shandong University, Jinan 250012, China

## Abstract

Oxidative stress and apoptosis play a vital role in the pathogenesis of contrast-induced acute kidney injury (CI-AKI). The purpose of our study was to investigate the protective effects and mechanisms of melatonin against CI-AKI in a CI-AKI mouse model and NRK-52E cells. We established the CI-AKI model in mice, and the animals were pretreated with melatonin (20 mg/kg). Our results demonstrated that melatonin treatment exerted a renoprotective effect by decreasing the level of serum creatinine (SCr) and blood urea nitrogen (BUN), lessening the histological changes of renal tubular injuries, and reducing the expression of neutrophil gelatinase-associated lipid (NGAL), a marker of kidney injury. We also found that pretreatment with melatonin remarkably increased the expression of Sirt3 and decreased the ac-SOD2 K68 level. Consequently, melatonin treatment significantly decreased the oxidative stress by reducing the Nox4, ROS, and malondialdehyde (MDA) content and by increasing the superoxide dismutase (SOD) and glutathione peroxidase (GSH-Px) activity levels. The antiapoptotic effect of melatonin on CI-AKI was revealed by decreasing the ratio of Bax/Bcl2 and the cleaved caspase3 level and by reducing the number of apoptosis-positive tubular cells. In addition, melatonin treatment remarkably reduced the inflammatory cytokines of interleukin-1*β* (IL-1*β*), tumor necrosis factor *α* (TNF*α*), and transforming growth factor *β* (TGF*β*) in vivo and in vitro. Sirt3 deletion and specific Sirt3 siRNA abolished the above renoprotective effects of melatonin in mice with iohexol-induced acute kidney injury and in NRK-52E cells. Thus, our results demonstrated that melatonin exhibited the renoprotective effects of antioxidative stress, antiapoptosis, and anti-inflammation by the activation of Sirt3 in the CI-AKI model in vivo and in vitro. Melatonin may be a potential drug to ameliorate CI-AKI in clinical practice.

## 1. Introduction

With the increasing prevalence of cardiovascular diseases throughout the world, the use of contrast media (CM) in percutaneous coronary intervention (PCI) has increased as well. Hence, contrast-induced acute kidney injury (CI-AKI) has become a more common problem in clinical practice and is an important cause of hospital-acquired acute renal insufficiency [1–3] despite numerous attempts to reduce the incidence of CI-AKI, such as the improvement of hydration protocols and pretreatment with antioxidants, which are supposed to be less nephrotoxic. Although the exact pathophysiological mechanisms of CI-AKI are not fully understood, renal vasoconstriction with resultant renal medullary hypoxia and direct toxic effects of radiocontrast on renal tubules are two major mechanisms that have been found to be involved in the pathogenesis of CI-AKI [1, 2, 4]. In addition, the apoptosis and cell death of both renal epithelial and endothelial cells can result from CM due to the cytotoxicity caused by iodine [5].

Melatonin is mainly produced by the pineal gland and is well known for its natural antioxidant activity and free radical scavengers [6, 7]. Given its antioxidative, anti-inflammatory, and antiapoptotic properties, melatonin has the ability to protect against diseases. Published studies have elucidated that melatonin can contribute to the improvement of cardiac function in diabetic cardiomyopathy (DCM) [8, 9]. Recent studies have focused on the role of melatonin in the treatment of myocardial ischemia/reperfusion (MI/R) injury [10]. Other literature showed that melatonin has a renoprotective effect on arsenic-induced nephropathy [11]. However, the underlying mechanisms of how melatonin protects against CI-AKI remain poorly understood.

Sirtuin-3 (Sirt3), a highly conserved nicotinamide adenine dinucleotide (NAD^+^)-dependent histone deacetylase, is mainly localized in the mitochondria and is highly expressed in the kidney [12]. Sirt3 plays a vital role in maintaining mitochondrial function. One study showed that it acts as the primary deacetylase for mitochondrial target proteins by deacetylating key lysine residues [13]. One of our previously published studies demonstrated that Sirt3 protects against chronic kidney injury by deacetylating KLF15 [14]. Morigi et al. [15] found that Sirt3 improves mitochondrial dynamics to protect against acute kidney injury (AKI). Another study found that Sirt3 attenuates tubular epithelial cell apoptosis, oxidative stress, and mitochondrial dysfunction in cisplatin-induced AKI [16]. One of our more recent studies showed that endogenous Sirt3 plays a protective effect in CI-AKI models [17]. However, the specific mechanisms of how Sirt3 exerts a protection on CI-AKI are unknown.

Therefore, the purpose of the current study is to evaluate the protective effect of melatonin on CI-AKI and the role of Sirt3 signaling in this process in vitro and in vivo.

Of note, our study also provides a basis for the prevention of CI-AKI with melatonin in future clinical practice.

## 2. Methods and Materials

### 2.1. Animals and Treatment

The experiments were performed in accordance with the Animal Management Rules of the Chinese Ministry Health (Document No. 55, 2001) and were approved by the Animal Care and Use Committee of Shandong University. Sirt3 deletion (Sirt3^−/−^, 129-SIRT3tm1.1Fwa/J) mice were obtained from Jackson Laboratory (Bar Harbor, ME, USA), and wild-type (WT, 129S1/SvImJ) mice were purchased from the Department of Laboratory Animal Science of Peking University to serve as the control (Beijing, China). All mice were housed under standard conditions of 12 : 12-hr light-dark cycle and 50%–60% humidity at 22°C–24°C with food and water available. After one-week accommodation, the 8-week-old male Sirt3^−/−^ and WT mice were randomly assigned to different groups as experimental protocols.

In the first part of the animal study, the mice were divided into four groups (*n* = 10 in each group): (i) control group (Con), (ii) melatonin group (Mel), (iii) contrast media group (CM), and (iv) contrast media+melatonin group (CM+Mel). CI-AKI was induced in mice as described previously with modifications [18, 19]. In brief, after water deprivation for 16 h and prior inhibition of prostaglandin and nitric oxide synthesis, mice in the CM group received low-osmolar monomeric iodinated radiocontrast media iohexol (Omnipaque, 3.0 g iodine/kg) through a tail vein. For inhibition of nitric oxide synthase and cyclooxygenase, mice were injected with indomethacin (10 mg/kg i.p., dissolved in dimethyl sulfoxide) and NG-nitro-L-arginine methyl ester (L-NAME, 10 mg/kg i.p., dissolved in 0.9% normal saline) 15 min before iohexol administration. Melatonin was initially dissolved in absolute ethanol and then diluted in sterile water to a final concentration of 4% ethanol. The mice received melatonin (20 mg/kg i.p.) 30 min before CM injection. Controls received an injection of saline alone at each time point. After iohexol injection, the mice were then given free access to water and food.

In the second part of the animal study, to investigate the effects of melatonin treatment on Sirt3 signaling, we established Sirt3^−/−^ mice and divided them into four groups (*n* = 10 in each group): (i) WT+CM, (ii) Sirt3^−/−^+CM, (iii) WT+CM+Mel, and (iv) Sirt3^−/−^+CM+Mel. All the animals were euthanized after 24 h using an overdose of sodium pentobarbital (1%). Blood samples were collected to isolate serum and then stored in a -80°C freezer. The left kidney was harvested for molecular assessments. The right kidney was fixed in 4% formalin for histological measurements.

### 2.2. Serum Measurements

The levels of serum creatinine (SCr) and blood urea nitrogen (BUN) were determined using an automatic analyzer (Roche, Basel, Switzerland) for the measurements of renal function.

### 2.3. Cell Culture

In our study, Rat NRK-52E (normal rat proximal tubular epithelial cell line) was obtained from the Type Culture Collection of the Chinese Academy of Sciences (Shanghai, China). Cells were cultured in six-well dishes and were grown in RPMI-1640 medium supplemented with 10% fetal bovine serum (Sigma) plus 1% streptomycin/penicillin (Solarbio) at 37°C in a 5% CO_2_ atmosphere. Medium was replaced every 2–3 days. When the cells reached 80% confluence in culture wells, cells were randomly divided into the following groups: (i) control: the cells were treated with serum-free medium; (ii) Mel: the cells were treated with melatonin (100 *μ*mol/L) for 1 h [8]; (iii) iohexol: the cells were subjected to iohexol (100 mgI/mL) for 4 h [20]; (iv) iohexol+Mel: the cells were pretreated with melatonin (100 *μ*mol/L) for 1 h and then subjected to iohexol (100 mgI/mL) for 4 h; (v) si-Sirt3+iohexol: the cells were pretreated with Sirt3 siRNA and then subjected to iohexol (100 mgI/mL) for 4 h; (vi) si-Sirt3+iohexol+Mel: the cells were pretreated with Sirt3 siRNA, incubated with medium supplemented with melatonin (100 *μ*mol/L) for 1 h and, then, subjected to iohexol (100 mgI/mL) for 4 h. For the Sirt3 silencing experiments in vitro, the NRK-52E cells were transfected with 50 nM of small interfering RNA (siRNA) specific for Sirt3 with Lipofectamine 2000 (Invitrogen, Waltham, MA, USA) following the manufacturer's instructions for 24 h. The sequence of siRNA-Sirt3 was (5′-3′) GCGUUGUGAAACCUGACAUTTAUGUCAGGUUUCACAACGCTT.

### 2.4. ROS Measurement

The level of intracellular reactive oxygen species (ROS) was measured using 2′,7′-dichlorofluorescein diacetate (DCFH-DA, Beyotime, Jiangsu, China) test kit according to the manufacturer's instructions. In brief, the experimental group cells were preincubated with/without melatonin (100 *μ*mol/L) for 1 h and followed by further incubation in iohexol (100 mgI/mL). After 4 h, cells were treated with DCFH-DA (10 *μ*mol/L) incubation in the dark for 40 min at 37°C. Then, cells were washed three times with PBS to remove any remaining DCFH-DA. The ROS generation was detected by a fluorescence microscopy (488 nm filter, Olympus, Tokyo, Japan).

### 2.5. RNA Isolation and Real-Time Quantitative PCR (RT-PCR)

Total RNA was extracted from excised kidney tissues using TRIzol ® reagent (Invitrogen Life Technologies, Carlsbad, CA, USA) and cells using RNA fast200 (Fastagen, Shanghai, China) in accordance with the manufacturer's instructions. Using the PrimeScript RT reagent kit (Takara Bio, Kusatsu, Japan), we synthesized cDNA from 1 *μ*g of total RNA. Relative quantitative real-time PCR was performed on the device of Light Cycler 480 system (Roche Diagnostics), and SYBR Green Master Mix (Takara Bio) was used following the protocol of the manufacturers. The data were calculated based on the threshold cycle values (Ct) and relative mRNA expression levels. The primer sequences were shown in Table S1.

### 2.6. Determination of MDA, SOD, and GSH-Px

The malondialdehyde (MDA) content and the superoxide dismutase (SOD) and glutathione peroxidase (GSH-Px) activity levels in kidney tissues were assayed using commercial kits (Jiancheng Biotech, Nanjing, China) following the instructions of the respective manufacturers. The data were analyzed spectrophotometrically using a SpectraMax M5 (Molecular Devices, San Jose, CA, USA).

### 2.7. Histology and Immunochemistry Staining

The kidney tissues were dehydrated and embedded in paraffin. After embedding in paraffin, 5 *μ*m sections were stained with hematoxylin and eosin (HE) to evaluate renal morphology. Histopathology changes including tubular epithelial cell swelling, intertubular hemorrhaging, brush border loss, cytoplasmic vacuolar degeneration, and intratubular cast formation were evaluated by the degree of tubular injury graded from 0 to 4 as described previously [21]. The data analysis was conducted by two members of our team, who were blinded to the experimental groups.

The slides were incubated overnight at 4°C with the primary antibodies against Neutrophil gelatinase-associated lipocalin (NGAL, BOSTER, PB9609), Nox4 (Proteintech, 14347-1-AP), Bax (Abcam, ab32503), Bcl2 (Abcam, ab182858), and cleaved caspase3 (Cell Signaling Technology, #9664) for immunohistochemistry. The next day, horseradish peroxidase-conjugated secondary antibodies were added for 30 min at 37°C. The sections were washed with PBS, and diaminobenzidine (ZSGB-Bio, Beijing, China) was added for color development. In addition, nuclei were counterstained with hematoxylin. The analysis was carried out using the ImageJ software (NIH Image, Bethesda, MD, USA).

### 2.8. TUNEL Assay

The TdT-mediated dUTP nick end labeling (TUNEL) assay was performed to measure renal tubular epithelial cell death using the In Situ Cell Death Detection kit (Roche Molecular Biochemicals, Mannheim, Germany) according to the manufacturer's instructions. The kidney frozen sections were hydrated in PBS and subsequently immersed in permeabilization solution (0.1% Triton X-100) for 3 min. 4′,6-diamidino-2-phenylindole (DAPI) was selected to stain nuclei after TUNEL reaction. The apoptotic ratio was expressed as the number of positive cells to total cells.

### 2.9. Western Blot Analysis

Protein was extracted from the kidney tissues and NRK-52E cells using RIPA buffer containing protease inhibitor cocktail. Then, the protein concentration was quantified by Pierce BCA Protein Assay Kit (Beyotime, Jiangsu, China). Equal amounts of protein were separated through 10% or 12% SDS-PAGE before being electrotransferred onto PVDF membranes (Millipore, Billerica, MA, USA). Each membrane was blocked with 5% fat-free milk for 1 h at room temperature and then incubated overnight at 4°C with specific primary antibodies against Sirt3 (Cell Signaling Technology, #5490); cleaved caspase3, Nox4, and GAPDH (Proteintech, 60004-1-Ig); ac-SOD2 K68 (Abcam, ab137037), and Bax and Bcl2 (Proteintech, 60178-1-Ig). Then, the membranes were washed three times in TBST and incubated with appropriate secondary antibodies for 1 h at room temperature. The blots were then visualized using enhanced chemiluminescence reagents (Millipore).

### 2.10. Statistical Analysis

All data were presented as mean ± standard error of the mean (SEM), and the statistical analyses were administrated using Graphpad Prism 8.0.1 (Graphpad Software, Inc., California, USA). Differences between the two groups were evaluated by student's *t*-test and between multiple groups by one-way ANOVA. Differences with *P* < 0.05 were regarded as statistically significant.

## 3. Results

### 3.1. Melatonin Improved Renal Function, and Attenuated Morphological Injury of Kidney following CM Administration in Mice

**Figure 1:**
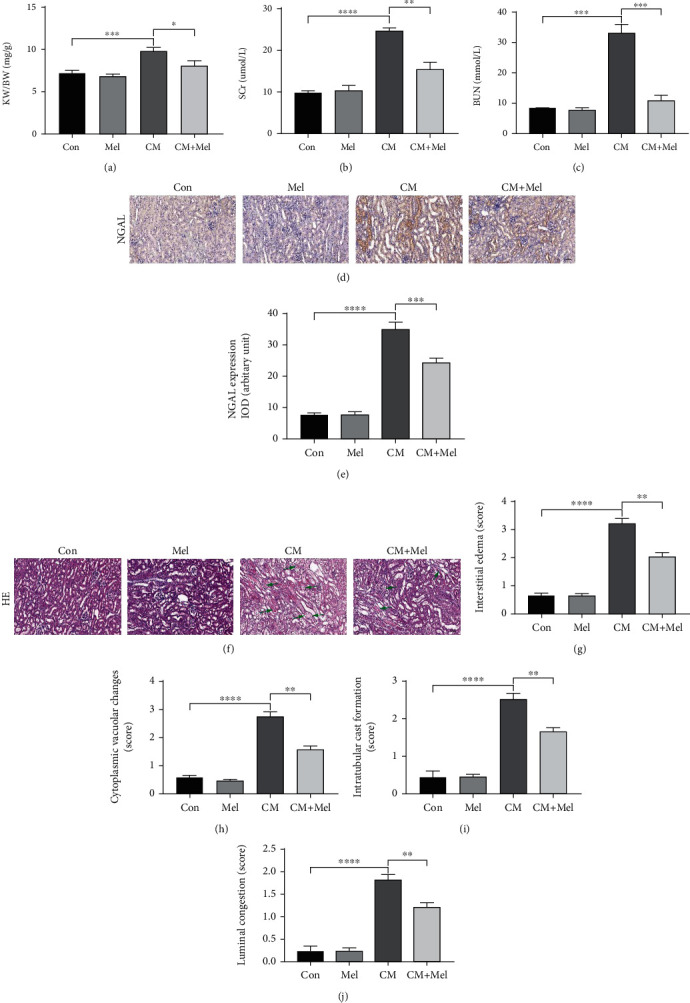
Melatonin administration ameliorated renal function and reduced the expression of NGAL, renal tubular injure score after iohexol-treatment in vivo. (a) The ratio of KW to BW in different groups. (b, c) The level of SCr and BUN in different groups. (d) Representative immunohistochemical staining of NGAL. (e) Quantification of NGAL immunostaining. (f) Representative micrographs of HE-stained of kidney on morphological changes. (g–j) Semiquantitative analysis of interstitial edema, cytoplasmic vacuolar changes, intratubular cast formation, and luminal congestion to evaluated renal tubular injures. KW: kidney weight. BW: body weight. SCr: serum creatinine. BUN: blood urea nitrogen. NGAL: neutrophil gelatinase-associated lipid. HE: hematoxylin and eosin staining. All experiments were calculated at least 3 times. Data are presented as the means±SEM. ^∗^*P* < 0.05, ^∗∗^*P* < 0.01, ^∗∗∗^*P* < 0.001, ^∗∗∗∗^*P* < 0.001.

To investigate the effect of melatonin on CI-AKI, we firstly measured the body weight and kidney weight and found that the ratio of kidney weight to body weight (KW/BW) was significantly decreased in the CM+Mel group compared to the CM group (Figure 1(a)). As shown in Figures 1(b) and 1(c), both the SCr and BUN levels were remarkably increased in the CM group compared with the control group. In the CM+Mel group, the administration of melatonin significantly decreased the SCr and BUN levels compared with those in the CM group. Immunohistochemistry findings showed that NGAL, a novel biomarker for the early detection of CI-AKI, was predominantly expressed in the cytoplasm of proximal tubular cells (Figure 1(d)). Compared with the control group, the expression of NGAL was significantly increased in the CM group and decreased with the treatment of melatonin in the CM+Mel group (Figure 1(e)). The pathological findings with HE staining of kidney sections in all groups are shown in Figure 1(f). Injuries including interstitial edema, cytoplasmic vacuolar changes, intratubular cast formation, and luminal congestion in the renal tubular were observed in the CM mice. In the CM+Mel group, pretreatment with melatonin evidently alleviated the development of these lesions and tissue damage (Figures 1(g)–1(j)). These results demonstrated that melatonin played a protective effect against CI-AKI.

### 3.2. Melatonin Elevated Sirt3 Expression and Activity, Exerted a Protective Effect against Oxidative Stress and Inflammation Induced by CM

**Figure 2:**
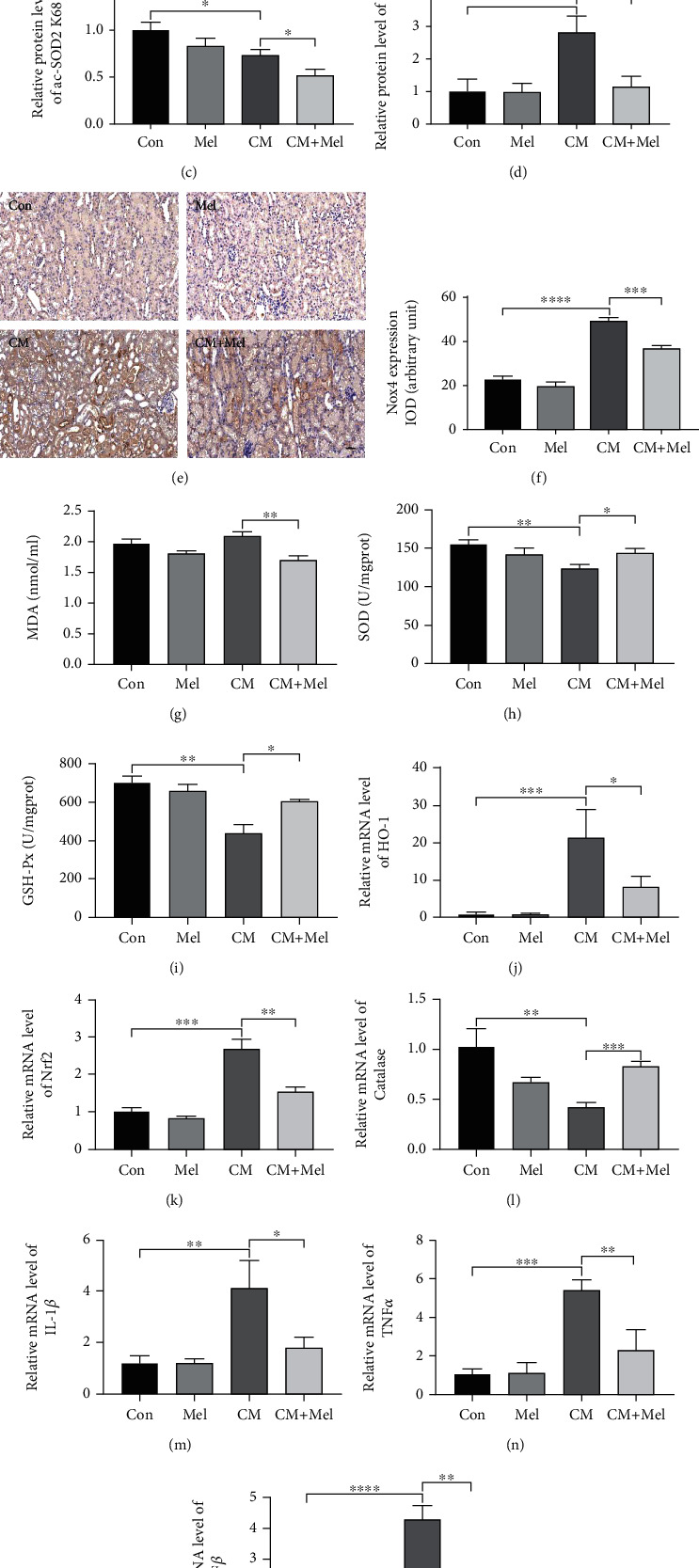
Melatonin decreased kidney oxidative stress and inflammation caused by iohexol in vivo. (a) Representative western blots. (b–d) Quantitative analysis of protein expression of Sirt3, ac-SOD2 K68, and Nox4. (e) Representative immunohistochemical staining of Nox4. (f) Quantitative analysis of Nox4 immunostaining. (g) Renal MDA content. (h) Renal SOD activity. (i) Renal GSH-Px activity. (j–l) Quantitative analysis of HO-1, Nrf2, and Catalase mRNA expression in the kidney of four groups. (m–o) Quantitative analysis of IL-1*β*, TNF*α*, and TGF*β* mRNA expression in the kidney of four groups. MDA: malondialdehyde. SOD: superoxide dismutase. GSH-Px: glutathione peroxidase. All experiments were repeated at least 3 times. Data are expressed as mean ± SEM. ^∗^*P* < 0.05, ^∗∗^*P* < 0.01, ^∗∗∗^*P* < 0.001, ^∗∗∗∗^*P* < 0.001.

Firstly, we measured the protein expression of Sirt3 and ac-SOD2 K68, a marker for Sirt3 activity. Our results indicated that the level of Sirt3 was upregulated in the CM group compared with the control group. The strongest level of Sirt3 was observed in the mice pretreated with melatonin, and the level of ac-SOD2 K68 was predominantly decreased (Figures 2(a)–2(c)). Nox4 plays an important role in CM-stimulated superoxide production and contributes to oxidative stress. We found that the mice in the CM group had increased Nox4 levels, but pretreatment with melatonin in the CM+Mel group alleviated this tendency (Figures 2(a) and 2(d)–2(f)). Furthermore, the MDA content was increased, and the activities of SOD and GSH-Px were decreased in the kidney tissues of the CM group, showing that oxidative stress occurred following CM administration. However, melatonin treatment decreased the MDA content and increased the antioxidative capacity by upregulating the activities of SOD and GSH-Px (Figures 2(g)–2(i)). To further explore the antioxidative effects of melatonin, we also detected the gene expressions of Heme Oxygenase-1 (HO-1), nuclear factor E2-related factor 2 (Nrf2), and Catalase. Compared with the control group, the mRNA levels of HO-1and Nrf2 were significantly increased and the level of Catalase was decreased in the CM group (Figures 2(j)–2(l)). However, these observations were reversed in mice pretreated with melatonin. To elucidate the protection of melatonin on inflammation, inflammatory cytokines of interleukin-1*β* (IL-1*β*), tumor necrosis factor *α* (TNF*α*), and transforming growth factor *β* (TGF*β*) were measured. We found that the mRNA level of these inflammatory cytokines was lower in the CM+Mel group compared with the CM group (Figures 2(m)–2(o)). Altogether, melatonin's renoprotective effect may be enacted through the activation of Sirt3.

### 3.3. Melatonin Upregulated Bcl2 Expression Level and Decreased Bax, Cleaved Caspase-3 Expression Levels, and Apoptotic Ratio following CM

**Figure 3:**
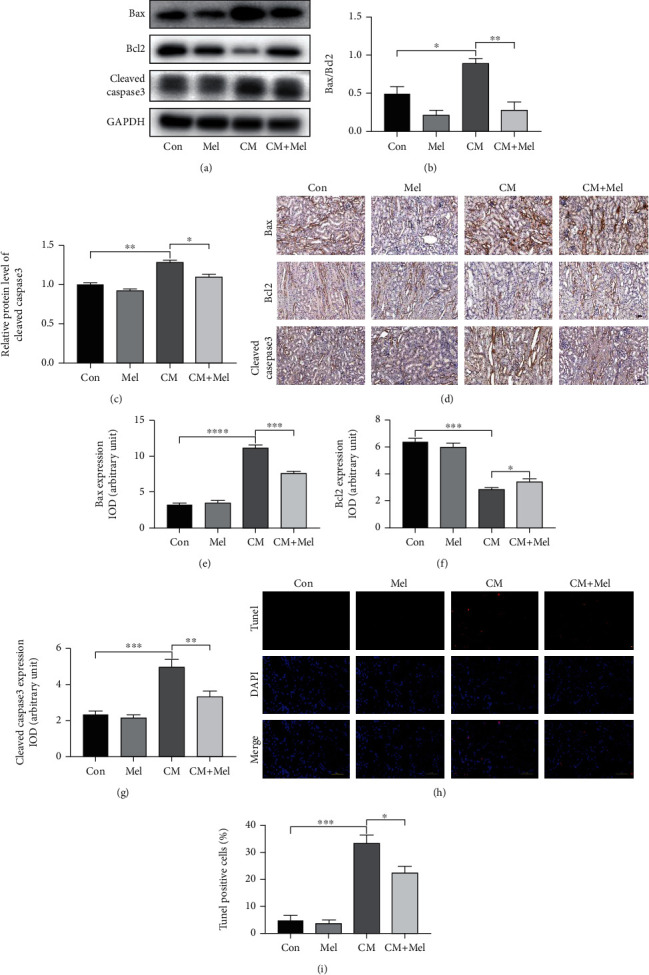
Effect of melatonin treatment on antiapoptosis after iohexol administration in vivo. (a) Representative western blots. (b, c) Quantitative analysis of the ratio of Bax to Bcl-2 and the expression of cleaved caspase3 were examined. (d) Representative immunohistochemical staining of Bax, Bcl2, and cleaved caspase3. (e–g) Quantification of Bax, Bcl2, and cleaved caspase3. (h) Representative digital images of apoptotic cells. The apoptotic cells were detected by TUNEL (red), and the nuclei were detected by DAPI (blue). (i) Percentage of TUNEL-positive nuclei. All experiments were repeated at least 3 times. Data are presented as mean ± SEM. ^∗^*P* < 0.05, ^∗∗^*P* < 0.01, ^∗∗∗^*P* < 0.001, ^∗∗∗∗^*P* < 0.001.

To further confirm the renal protection effect of melatonin against CI-AKI, we evaluated the expression of proapoptotic and antiapoptotic proteins in our study. The results indicated that the level of cleaved caspase3 as well as the ratio of Bax/Bcl2 markedly elevated in the mice subjected to iohexol treatment, and the effect was inhibited by pretreatment with melatonin (Figures 3(a)–3(c)). Similar results were observed in immunohistochemistry staining assay (Figures 3(d)–3(g)). Moreover, iohexol caused a remarkable increase in cell apoptosis, as observed by the increased apoptotic ratio compared with the control group. Treatment with melatonin significantly blocked the increase in apoptotic index caused by iohexol (Figures 3(h) and 3(i)). In summation, melatonin treatment decreased apoptosis in the kidney of iohexol-treated mice.

### 3.4. The Sirt3 Signaling Pathway Is Involved in the Renoprotective Effects of Melatonin in CI-AKI Model

**Figure 4:**
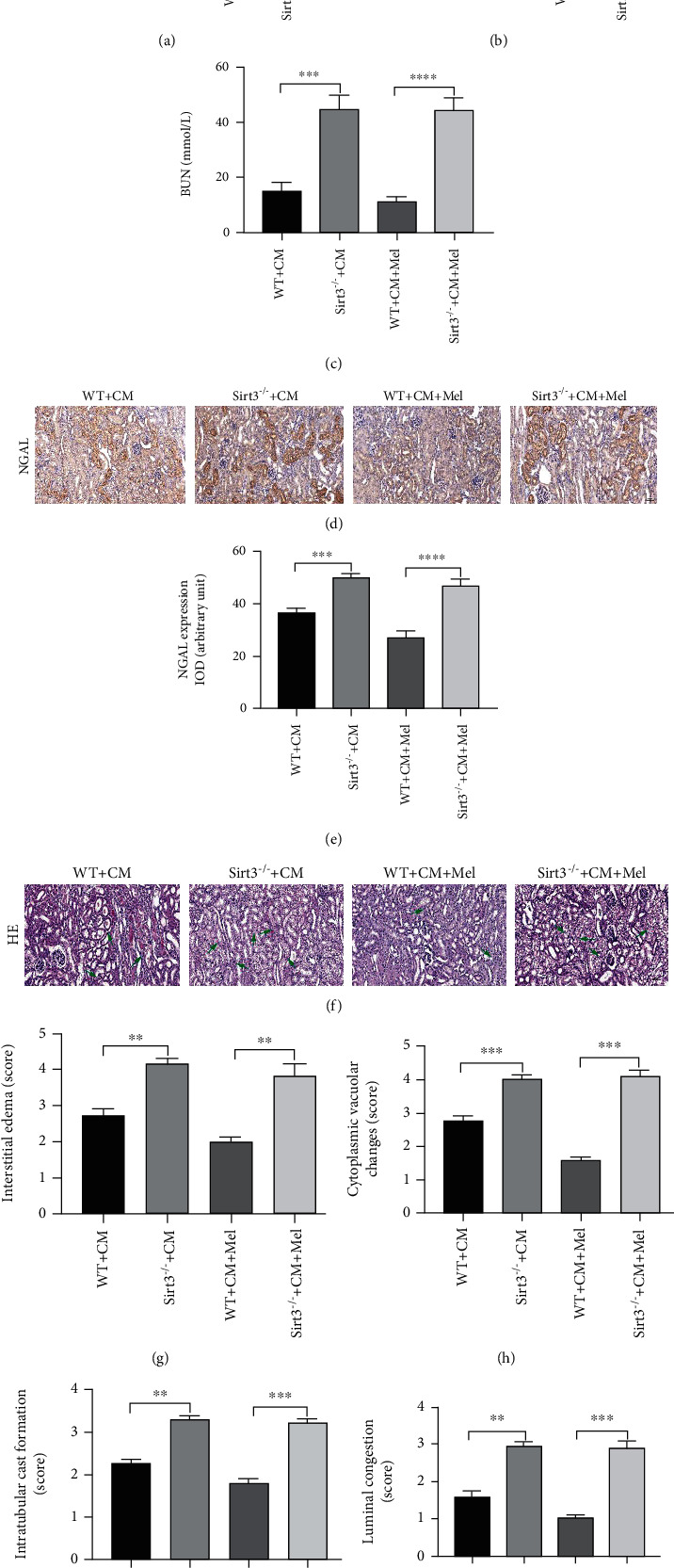
Sirt3 deficiency abolished the renoprotection of melatonin by deteriorating renal function and increasing renal tubular injure score in vivo. (a) The ratio of KW to BW in four groups. (b, c) The level of SCr and BUN in four groups. (d, e) Representative immunohistochemical staining and quantitative analysis of NGAL. (f) Representative micrographs of HE-stained kidney on morphological changes. (g–j) Semiquantitative analysis of interstitial edema, cytoplasmic vacuolar changes, intratubular cast formation, and luminal congestion to evaluated renal tubular injures. All experiments were performed at least 3 times. Data are expressed as mean ± SEM. ^∗^*P* < 0.05, ^∗∗^*P* < 0.01, ^∗∗∗^*P* < 0.001, ^∗∗∗∗^*P* < 0.001.

Sirt3 deletion mice were used to elucidate whether the Sirt3 signaling pathway is involved in the protective effects of melatonin. Our results indicated that the ratio of KW/BW, the levels of SCr and BUN, and the expression of NGAL were remarkably increased in the Sirt3^−/−^+CM group compared with the WT+CM group. However, in the Sirt3 deletion mice subjected to iohexol and pretreated with melatonin, these changes were not affected (Figures 4(a)–4(e)). The pathological findings with HE staining of the kidney on morphological changes were examined as well. The results demonstrated that the Sirt3 deletion mice with iohexol treatment developed deteriorated renal tubular damage, and the protective effect of melatonin was abolished in Sirt3 deletion mice unlike in WT mice (Figures 4(f)–4(j)). These findings showed that endogenous Sirt3 is a prerequisite for the renoprotective effect of melatonin in the CI-AKI model.

### 3.5. The Sirt3 Signaling Pathway Participates in the Antioxidative and Anti-Inflammatory Effects of Melatonin in Mice

**Figure 5:**
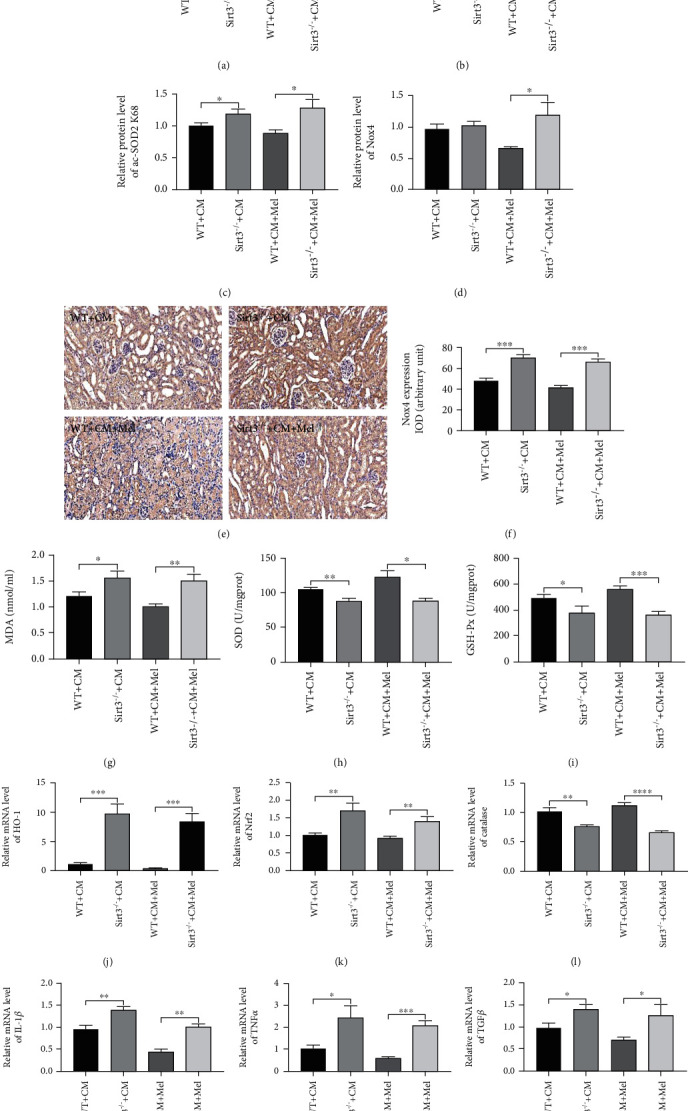
Sirt3 deficiency abolished the antioxidative effects of melatonin following iohexol. (a) Representative western blots. (b) Sirt3 expression. (c) ac-SOD2 K68 expression. (d) Nox4 expression. (e, f) Representative immunohistochemical staining and quantitative analysis of Nox4. (g) Renal MDA content. (h) Renal SOD activity. (i) Renal GSH-Px activity. (j–l) Quantitative analysis of HO-1, Nrf2, and Catalase mRNA expression in the kidney of different groups. (m–o) Quantitative analysis of IL-1*β*, TNF*α*, and TGF*β* mRNA expression in the kidney of different groups. All experiments were performed at least 3 times. Data are presented as mean ± SEM. ^∗^*P* < 0.05, ^∗∗^*P* < 0.01, ^∗∗∗^*P* < 0.001, ^∗∗∗∗^*P* < 0.001.

Our findings demonstrated that melatonin decreased ac-SOD2 K68 expression in WT mice, but this effect was diminished in Sirt3 deletion mice (Figures 5(a)–5(c)). Western blot and immunostaining showed that the expression of Nox4 was increased in Sirt3 deletion mice, and the protective effects of melatonin on antioxidative stress were impaired by Sirt3 deletion (Figures 5(a) and 5(d)–5(f)). In addition, melatonin-induced reduction of MDA content was largely inhibited by Sirt3 deletion, and the upregulated antioxidative capacity (increased activities of SOD and GSH-Px) induced by melatonin was also weakened by Sirt3 deletion (Figures 5(g)–5(i)). Furthermore, the mRNA levels of HO-1 and Nrf2 were increased, and Catalase was decreased in Sirt3 deletion mice compared with WT mice those subjected to iohexol. While, melatonin failed to exhibit the antioxidative stress effect in Sirt3 deletion mice compared with WT mice in the CI-AKI model (Figures 5(j)–5(l)). Similarly, the inflammatory cytokines of IL-1*β*, TNF*α*, and TGF*β* significantly increased in Sirt3 deletion mice compared with WT mice, and the anti-inflammatory effect of melatonin was reversed by Sirt3 deletion mice (Figures 5(m)–5(o)). These data indicated that Sirt3 deletion deteriorated oxidative stress and inflammation in the CI-AKI model and the renoprotective effect of melatonin by activating the Sirt3 signaling pathway.

### 3.6. The Sirt3 Signaling Pathway Participates in the Antiapoptotic Effects of Melatonin in the CI-AKI Model

**Figure 6:**
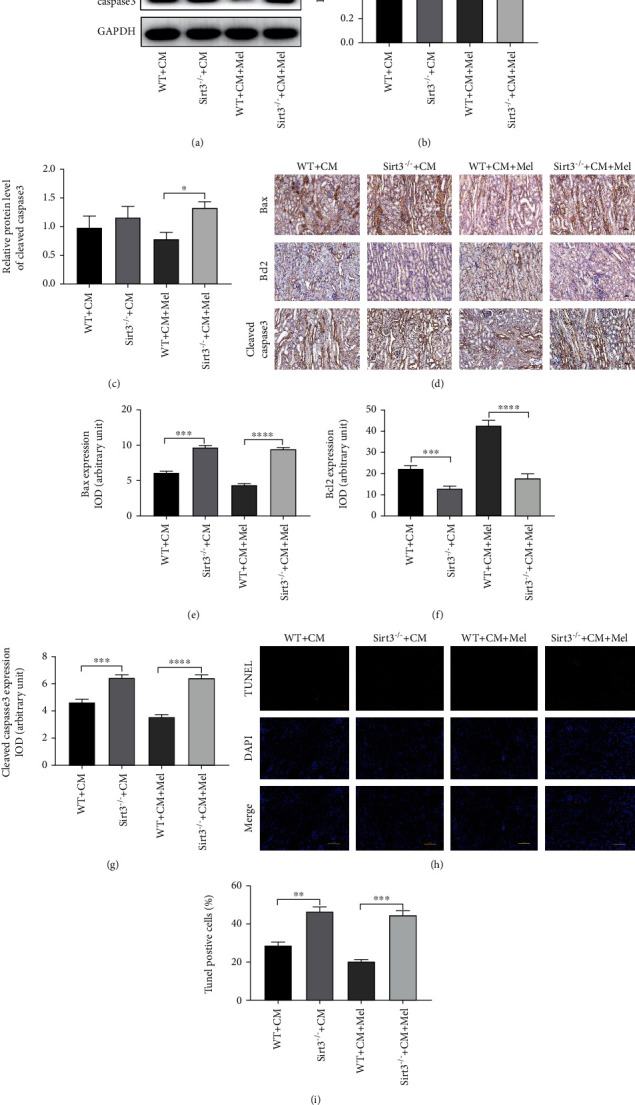
Sirt3 deficiency abolished the antiapoptotic effects of melatonin following iohexol. (a) Representative western blots. (b) Quantitative analysis of the ratio of Bax to Bcl-2. (c) Quantification of the expression of cleaved caspase3. (d) Representative immunohistochemical staining of Bax, Bcl2, and cleaved caspase3. (e–g) Quantification of Bax, Bcl2, and cleaved caspase3. (h) Representative micrographs of apoptotic cells. The apoptotic cells were detected by TUNEL (red), and the nuclei were detected by DAPI (blue). (i) Percentage of TUNEL-positive nuclei. All experiments were performed at least 3 times. Data are expressed as mean ± SEM. ^∗^*P* < 0.05, ^∗∗^*P* < 0.01, ^∗∗∗^*P* < 0.001, ^∗∗∗∗^*P* < 0.001.

Our results indicated that Sirt3 deletion notably abolished the antiapoptotic effect of melatonin by increasing the ratio of Bax/Bcl2 and the expression of cleaved caspase3 (Figures 6(a)–6(c)). Immunostaining also led to a similar conclusion (Figures 6(d)–6(g)). Moreover, reductions in apoptotic ratio caused by melatonin treatment were predominantly inhibited in Sirt3 deletion mice (Figures 6(h) and 6(i)). We also found that these above indexes were largely upregulated in Sirt3 deletion mice compared to WT mice. These data demonstrated that Sirt3 deletion significantly exacerbated apoptosis in the CI-AKI model, and the antiapoptotic effect of melatonin relies on the activation of the Sirt3-mediated signaling pathway.

### 3.7. Melatonin Treatment Mitigates Oxidative Stress, Apoptosis, and Inflammation in NRK-52E Cells following Iohexol

**Figure 7:**
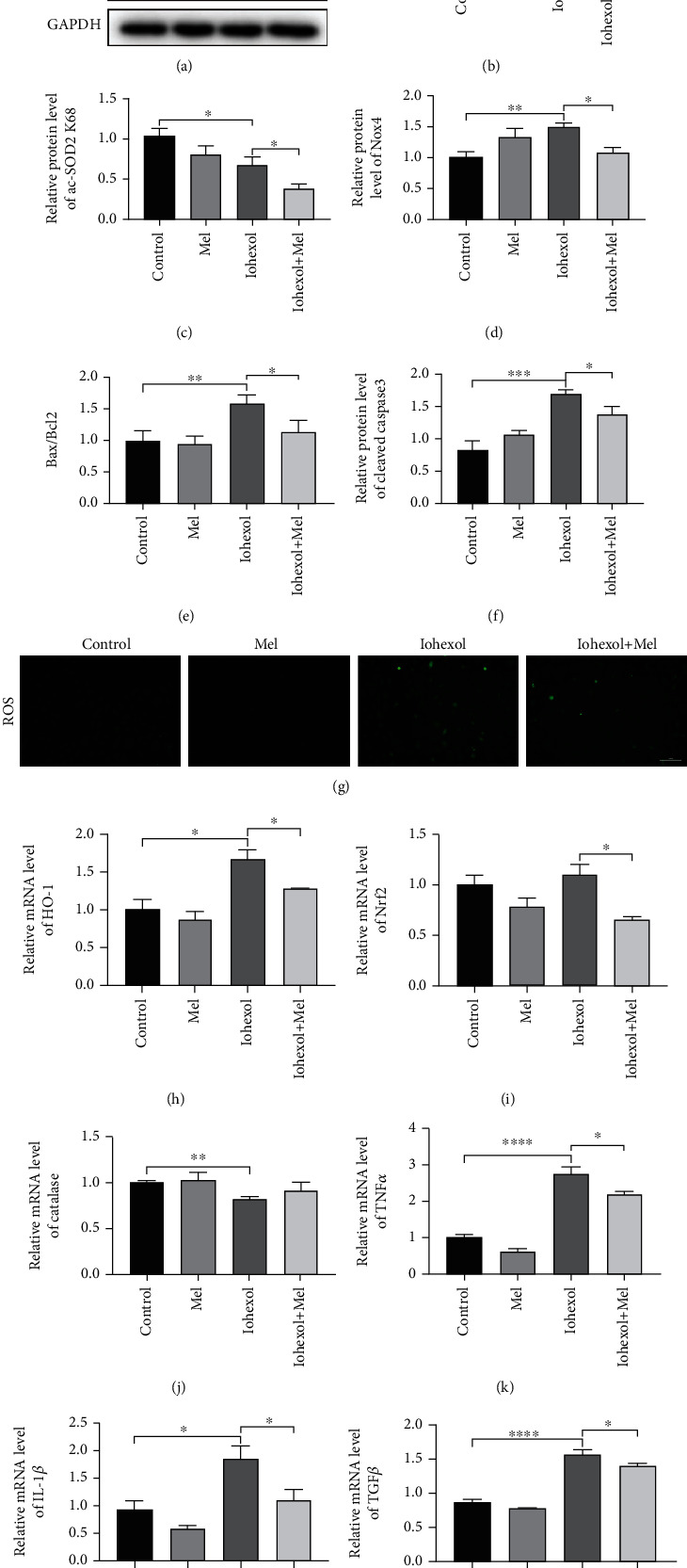
Effect of melatonin administration on oxidative stress, apoptosis, and inflammation in iohexol-injured NRK-52E cells in vitro. (a) Representative western blots. (b–f) Quantitative analysis of Sirt3, ac-SOD2 K68, Nox4, Bax/Bcl2, and cleaved-caspase3 expression. (g) ROS production. (h–j) Quantitative analysis of HO-1, Nrf2, and Catalase mRNA expression in NRK-52E cells of four groups. (k–m) Quantitative analysis of TNF*α*, IL-1*β*, and TGF*β* mRNA expression in NRK-52E cells of four groups. All experiments were repeated at least 3 times. Data are expressed as mean ± SEM. ^∗^*P* < 0.05, ^∗∗^*P* < 0.01, ^∗∗∗^*P* < 0.001, ^∗∗∗∗^*P* < 0.001.

NRK-52E cells were used to investigate whether melatonin directly protects against iohexol-induced injury and, if so, to illuminate the underlying mechanism. As shown in Figures 7(a) and 7(b), the expression of Sirt3 was upregulated with iohexol-stimulation, and the strongest level was observed in the iohexol+Mel group. Compared with the control group, iohexol induced a notable increase in ROS generation and Nox4 expression (Figures 7(a), 7(d), and 7(g)). Melatonin treatment also increased the activity of Sirt3 and alleviated the apoptosis (Figures 7(a)–7(c) and 7(e)–7(f)). Additionally, iohexol elevated the mRNA levels of HO-1 and Nrf2, accompanied by a reduction of Catalase (Figures 7(h)–7(j)). Iohexol also predominantly increased the mRNA levels of inflammatory cytokines on TNF*α*, IL-1*β*, and TGF*β* (Figures 7(k)–7(m)). However, preincubation with melatonin excessively reversed the alterations caused by iohexol. These results were consistent with our findings in vivo.

### 3.8. Sirt3 Plays a Major Role in the Antioxidative, Antiapoptotic, and Anti-Inflammatory Effects of Melatonin in NRK-52E Cells with Iohexol Treatment

**Figure 8:**
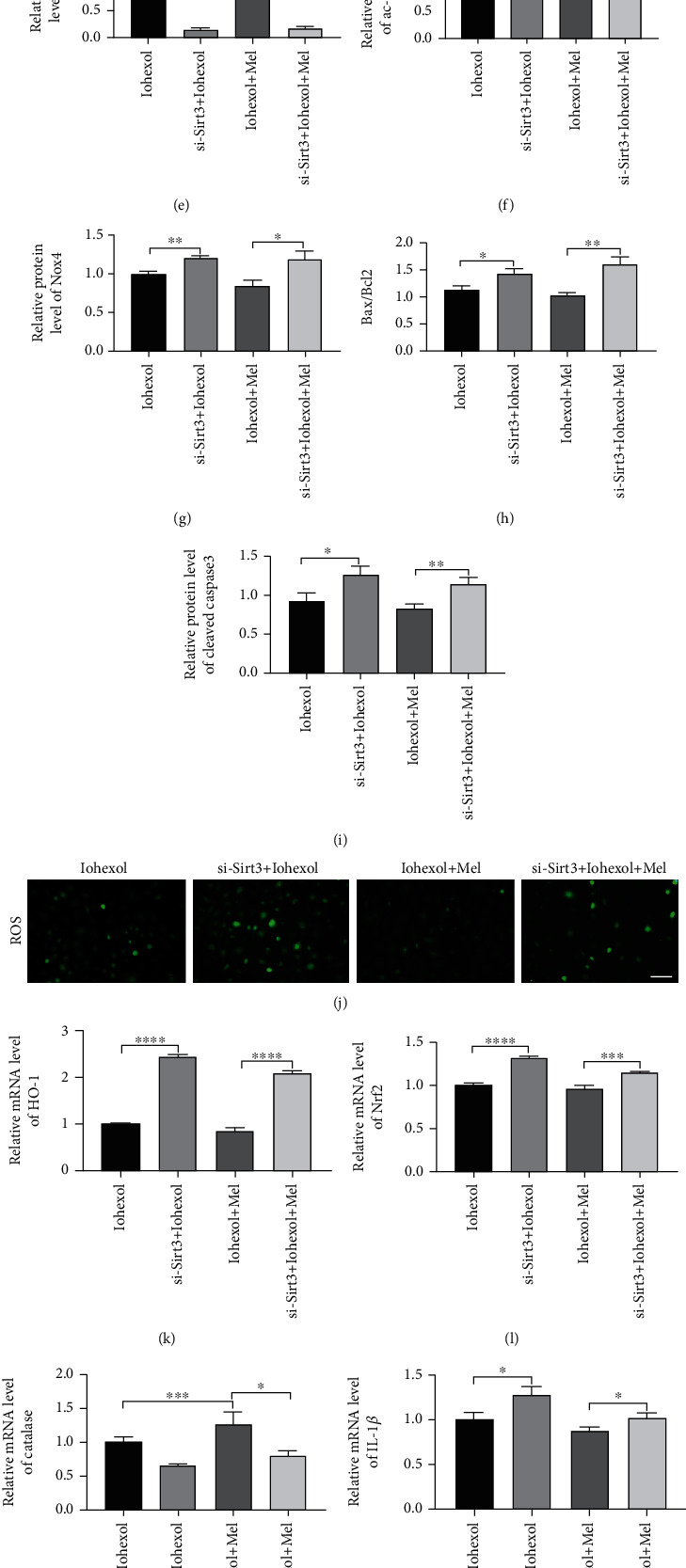
Sirt3 siRNA abolished the antioxidative, antiapoptotic, and anti-inflammatory effects of melatonin in iohexol-injured NRK-52E cells. (a–c) The knockdown efficacy of Sirt3 siRNA in NRK-52E cells. (d) Representative western blots. (e–i) Quantitative analysis of Sirt3, ac-SOD2 K68, Nox4, Bax/Bcl2, and cleaved-caspase3 expression. (j) ROS production. (k–m) Quantitative analysis of HO-1, Nrf2, and Catalase mRNA expression in NRK-52E cells of four groups. (n–p) Quantitative analysis of TNF*α*, IL-1*β*, and TGF*β* mRNA expression in NRK-52E cells of four groups. All experiments were calculated at least 3 times. Data are expressed as mean ± SEM. ^∗^*P* < 0.05, ^∗∗^*P* < 0.01, ^∗∗∗^*P* < 0.001, ^∗∗∗∗^*P* < 0.001.

Firstly, we evaluated the knockdown efficiency of Sirt3 siRNA in NRK-52E cells and found that the level of Sirt3 was predominantly decreased by Sirt3 siRNA (Figures 8(a)–8(c)). The effect of melatonin on Sirt3 expression was significantly blocked by Sirt3 siRNA (Figures 8(d)–8(f)). Sirt3 siRNA significantly decreased the antioxidative effect of melatonin by increasing Nox4 expression and ROS production (Figures 8(d), 8(g), and 8(j)). The similar conclusion was observed in apoptotic level (Figures 8(d) and 8(h)–8(i)). Sirt3 siRNA also elevated the mRNA levels of HO-1 and Nrf2, which was accompanied by a decrease of Catalase caused by melatonin (Figures 8(k)–8(m)). In addition, Sirt3 siRNA increased the mRNA levels of inflammatory cytokines on TNF*α*, IL-1*β*, and TGF*β* compared with the iohexol+Mel group (Figures 8(n)–8(p)). Compared with the iohexol group, the administration of Sirt3 siRNA in the si-Sirt3+iohexol group exacerbated the oxidative stress and inflammation induced by iohexol. These findings indicated that Sirt3 siRNA largely abolished the renoprotective effect of melatonin.

## 4. Discussion

In clinical practice, CI-AKI is an important complication in diagnostic and interventional procedures requiring the use of iodinated contrast media [22], CI-AKI may increase the costs of medical care and prolong hospital stays [23], and it is related to the accelerated progression of chronic kidney disease (CKD) [24]. It is a preventable cause of hospital-acquired renal failure, and prophylactic hydration therapy is widely used to relieve CI-AKI. However, the use of elderly, hypertension or heart and renal insufficiency is limited and may be “much ado about nothing” in most patients [25]. In addition, the mechanisms of CI-AKI are unknown, and effective drugs for protecting against CI-AKI are not available. Therefore, the development of novel strategies to prevent CI-AKI remains an urgent and challenging task.

In our study, the major findings are as follows: (i) melatonin protects against iohexol-induced AKI in the kidney (in vivo) and NRK-52E cells (in vitro), and (ii) melatonin effectively attenuates oxidative stress, apoptosis, and inflammation caused by iohexol via activation of Sirt3. In the present study, we investigated the effects of melatonin treatment on CI-AKI in mice. Our results demonstrated that administration of iohexol contributed to AKI, which was characterized by deteriorated renal function and renal tubular injury. Melatonin administration before iohexol offered renoprotective effects for the kidney and NRK-52E cells, as confirmed by improved renal function lessened histopathological injury, decreased expression of NGAL, and reduced oxidative stress in kidney tissues and NRK-52E cells. The renoprotective effects of melatonin were confirmed by activation of Sirt3, as Sirt3 deletion mice and Sirt3 siRNA both remarkably diminished the protective effects of melatonin on iohexol treatment.

The specific mechanisms underlying CI-AKI are not fully illustrated, but previous studies have shown that oxidative stress plays an important role in the development of CI-AKI [21, 26]. Additionally, it is well known that Nox4 is the classic pathway mediating oxidative stress. In our research, the Nox4 level was remarkably increased after iohexol treatment, and a higher level was observed in Sirt3 deletion mice than in WT mice. The administration of CM also elevates ROS level, which can cause lipid peroxidation and changes in antioxidant enzyme activities, thus resulting in cytotoxic damage. In this study, a prominent increase in the MDA level and a decrease in the SOD and GSH-Px activities were detected in renal tissues of the iohexol group, and Sirt3 deletion aggravated these tendencies compared with WT mice. The results were in accordance with NRN-52E cells and our previous study [17]. Melatonin, due to its free radical scavenging and antioxidative functions, has been used effectively to ameliorate ischemia/perfusion injury [7–9]. Evidence has shown that the effects of melatonin in different conditions are mediated by modulating the expression and/or activity of sirtuins [27]. Among the sirtuins, Sirt3 is considered to be the most important deacetylase in mitochondria. In the present study, our findings indicated that iohexol treatment led to the increase of Sirt3, and melatonin administration before iohexol treatment caused a higher expression and activation of Sirt3, which was evaluated by the decrease of ac-SOD2 K68. The results were consistent with a previous study [8]. Previous literature has reported that melatonin exhibited a protective effect by reducing lipid oxidation in aristolochic acid-induced nephropathy in mice [28]. In our study, the protective effects of melatonin against CI-AKI were confirmed by the decrease of Nox4 expression, ROS production, and lipid peroxidation and the increase in SOD and GSH-Px activities in vivo and in vitro. Interestingly, Sirt3 deletion abolished the antioxidative effects of melatonin in the CI-AKI model. Therefore, melatonin exerts protective effects against iohexol-induced oxidative stress by increasing Sirt3 expression and activity.

Apoptosis is also responsible for the pathogenesis of AKI [29, 30]. An increasing number of studies have confirmed the antiapoptotic effects of melatonin for protecting against acute kidney ischemia/reperfusion injury [31]. In our study, we found that the ratio of Bax/Bcl2 and the expression of cleaved caspase3 were elevated following iohexol treatment in mice, which increased the number of TUNEL-positive cells in renal tubules, and melatonin treatment effectively reduced the apoptosis. Additionally, Sirt3 deletion abolished this beneficial effect of melatonin, as pretreatment with melatonin increased the expression of Sirt3 and remarkably ameliorated apoptosis-related molecular changes. These investigations suggested that Sirt3 deletion aggravated the rate of apoptosis in the CI-AKI model, and the protective effect of melatonin against apoptosis was mediated by the Sirt3 signaling pathway. Moreover, immunostaining pointed to a similar conclusion.

Previous data showed that melatonin protected against diabetic nephropathy through an anti-inflammatory effect [32, 33]. Combined with the present results, melatonin exhibited antioxidative and anti-inflammatory effects in both mice and NRK-52E cells treated with iohexol, and Sirt3 deletion reversed the beneficial effect of melatonin on anti-inflammation. However, the detailed molecular mechanism by which melatonin increases Sirt3 level and activity following iohexol administration remains unknown. Further studies focused on molecular mechanisms will be important for both understanding the pathogenesis of CI-AKI and evaluating melatonin's therapeutic potential.

## 5. Conclusions

In conclusion, our data demonstrated that melatonin exhibits renal protection in a CI-AKI model via antioxidative and antiapoptotic activity by activating the Sirt3 signaling pathway. These results are potentially important for considering melatonin treatment as an early therapeutic intervention to attenuate CI-AKI in patients with cardiovascular disease.

## Data Availability

The data used to support the findings of this study are available from the corresponding author upon request.

## Supplementary Materials

Supplementary Materials The primer sequences for qRT-PCR in our study see Table S1.
