# Supplementary material for: Melatonin Alleviates Contrast-Induced Acute Kidney Injury by Activation of Sirt3
Source: Oxid Med Cell Longev. 2021 May 25;2021:6668887. doi: 10.1155/2021/6668887 (PMC8169261; doi:10.1155/2021/6668887)
Supplement: Supplementary Materials — The primer sequences for qRT-PCR in our study see Table S1. [file 6668887.f1.docx]

**Supplementary Materials**

**Table S1** The primer sequences

| Gene | | Forward | Reverse |
| --- | --- | --- | --- |
| Mus-HO-1 | GACAGCCCCACCAAGTTCAA | | AGCTCCTCAAACAGCTCAATGT |
| Rattus-HO-1 | CTTCCCGAGCATCGACAACC | | AATGTTGAGCAGGAAGGCGG |
| Mus-Nfe2l2 | CCCAGCAGGACATGGATTTGA | | AGCTCATAGTCCTTCTGTCGC |
| Rattus-Nrf2 | ATTCCCAGCCACGTTGAGAG | | TCCTGCCAAACTTGCTCCAT |
| Mus-TNFα | AGCCGATGGGTTGTACCTTG | | ATAGCAAATCGGCTGACGGT |
| Rattus-TNFα | ATGGGCTCCCTCTCATCAGT | | GCTTGGTGGTTTGCTACGAC |
| Mus-IL1β | CTTCTTGGGACTGATGCTGGT | | CTCTGTGAAGTCTCCTCTCCG |
| Rattus-IL1β | GGGATGATGACGACCTGCTA | | ACAGCACGAGGCATTTTTGT |
| Mus-TGFβ | AGCTGCGCTTGCAGAGATTA | | AGCCCTGTATTCCGTCTCCT |
| Rattus-TGFβ | TGACATGAACCGACCCTTCC | | TGTGGAGCTGAAGCAGTAGT |
| Mus-Catalase | CCCCTATTGCCGTTCGATTCT | | TTCAGGTGAGTCTGTGGGTTT |
| Rattus-Catalase | CGATTCTCCACAGTCGCTGG | | GTTTCCCACAAGGTCCCAGT |
